# Vaccine Potential of Nipah Virus-Like Particles

**DOI:** 10.1371/journal.pone.0018437

**Published:** 2011-04-06

**Authors:** Pramila Walpita, Jennifer Barr, Michael Sherman, Christopher F. Basler, Linfa Wang

**Affiliations:** 1 Departments of Microbiology and Immunology, Center for Biodefense and Emerging Infectious Disease, University of Texas Medical Branch, Galveston, Texas, United States of America; 2 Australian Commonwealth Scientific and Industrial Research Organization, Australian Animal Health Laboratory, Geelong, Victoria, Australia; 3 Department of Biochemistry and Structural Biology, University of Texas Medical Branch, Galveston, Texas, United States of America; 4 Department of Microbiology, Mount Sinai School of Medicine, New York, New York, United States of America; University of Texas Medical Branch, United States of America

## Abstract

Nipah virus (NiV) was first recognized in 1998 in a zoonotic disease outbreak associated with highly lethal febrile encephalitis in humans and a predominantly respiratory disease in pigs. Periodic deadly outbreaks, documentation of person-to-person transmission, and the potential of this virus as an agent of agroterror reinforce the need for effective means of therapy and prevention. In this report, we describe the vaccine potential of NiV virus-like particles (NiV VLPs) composed of three NiV proteins G, F and M. Co-expression of these proteins under optimized conditions resulted in quantifiable amounts of VLPs with many virus-like/vaccine desirable properties including some not previously described for VLPs of any paramyxovirus: The particles were fusogenic, inducing syncytia formation; PCR array analysis showed NiV VLP-induced activation of innate immune defense pathways; the surface structure of NiV VLPs imaged by cryoelectron microscopy was dense, ordered, and repetitive, and consistent with similarly derived structure of paramyxovirus measles virus. The VLPs were composed of all the three viral proteins as designed, and their intracellular processing also appeared similar to NiV virions. The size, morphology and surface composition of the VLPs were consistent with the parental virus, and importantly, they retained their antigenic potential. Finally, these particles, formulated without adjuvant, were able to induce neutralizing antibody response in Balb/c mice. These findings indicate vaccine potential of these particles and will be the basis for undertaking future protective efficacy studies in animal models of NiV disease.

## Introduction

Since it was first recognized in 1998, Nipah virus (NiV) has caused several outbreaks in humans of encephalitic disease associated with high lethality. In the first outbreak, which was in Malaysia and Singapore, 265 humans became sick and some ∼40% of them died. Epidemiological links pointed to human contact with sick pigs in commercial piggeries, and the outbreak was brought under control through culling of approximately ∼1.1 million pigs [1], [2], [3], [4]. Since then, the virus has re-emerged in Bangladesh and neighboring India, starting in 2001, and between then and now, has caused several smaller but even deadlier disease outbreaks with case fatality rates ranging between 60 and 90% [5], [6], [7], [8]. Unlike the Malaysian outbreak, the route of transmission in these outbreaks was considered to be bat-to-human via food contaminated with bat saliva [9]. In some cases, nosocomial transmissibility and person-to-person spread was also noted [5], [10], [11], [12]. An additional concern is that NiV is also potentially an agent of agro-terror since the rate of transmission of this virus in the pig population is close to 100% [13]. Effective vaccine and therapies are needed to combat the threats posed by NiV.

NiV is a member of the genus *Henipavirus* in the subfamily *Paramyxovirinae*, family *Paramyxoviridae*. It has several distinctive genetic and biologic features [14], [15], [16], [17], [18] although its morphology and genome organization is similar to that of other members of the subfamily. NiV has six genes arranged in tandem, 3′-N, P, M, F, G and L-5′ [15], [16]. The N, P and L are required for reconstituting viral RNA polymerase activity, the matrix protein M is required for particle formation and budding, and the two surface glycoproteins G and F are required for attachment and entry into the host cell [19], [20]. EphrinB2 and B3 have been identified as the NiV entry receptors [21], [22], [23]. After fusion of the virus and the cell membrane, the viral ribonucleoprotein is released in to the cell cytoplasm. Following transcription and replication, the viral components migrate to the plasma membrane for assembly and budding of progeny particles [24], [25].

Two vaccination strategies for NiV disease prevention have already been explored experimentally: A canarypox virus-based vaccine vector approach was effective as veterinary vaccine [26], and it is in the process of further development. The same approach for human use vaccines is undergoing extensive evaluation, largely for HIV and AIDS [27]. A soluble NiV G protein approach has also shown promise [28], [29]. However, subunit approaches are in general less effective than particulate immunogens, and can suffer from suboptimal presentation to the immune system [30], [31], [32]. Immunogenicity in mice to NiV glycoproteins has been reported recently using two vectored approaches for gene delivery; one using Venezuelan equine encephalitis virus replicons [33] and the other involving inoculation of a mix of two complementing defective vesicular stomatitis virus (VSVΔG) vectors, one for expressing each of the two NiV glycoproteins [34]. The latter approach is new and seems promising but its regulatory approval as human vaccine might be problematic [34], [35].

In this study we have explored the potential of NiV virus-like particles (VLPs) as a vaccine. Plasmid-mediated expression of selected viral proteins results in the spontaneous assembly and release of VLPs. These particles make highly effective immunogens because they possess several features of the authentic virus such as their surface structure and dimensions [31], [36]. They are also safe because they do not contain any viral genetic material. VLPs, where one or more of the constituent proteins serve as immunogens (native VLPs), are particularly effective as vaccines for infectious disease. The fact that two such vaccines [Gardasil (Merck & Co) for human papillomavirus (HPV), and Sci-B-Vac (SciGen) and Bio-HepB (GlaxoSimthKline) for Hepatitis B virus (HBV)] have already been approved for human use, and many, for non-enveloped and enveloped viruses [31], [32], [37], [38], [39], [40], [41], [42] are at various stages of development, attests to the desirability of this approach for vaccine development.

The budding capacity of virus proteins as VLPs, the protein-protein interactions that facilitate this process, and the central role of M protein in VLP assembly and release has been described for several paramyxoviruses such as Sendai virus (SeV), Newcastle disease virus (NDV), respiratory syncytial virus (RSV), paramyxovirus simian virus 5 (PIV-5) and human parainfluenza virus type 1 (hPIV1) [20], [43], [44], [45], [46], [47]: The efficiency of VLP formation in virtually all these studies was based on M protein release in the supernatant. NiV virus-like particles have also been described [48]; the results of this study showed 1) that NiV G and F proteins individually retained some budding capacity although it was far less efficient than that of the M protein and 2) NiV N, M, F and G-containing VLPs resembled the virus in some respects but differed significantly from it with respect to ratio of VLP-incorporated F protein; most of it was present in precursor F_0_ form. Recently, the vaccine potential of native VLPs of NDV [49] has been described: these particles, composed of HN, F, M and NP proteins, had several virus-like properties. However, since the F protein in this formulation was modified by design to ablate the cleavage site, it remained in its precursor form; consequently, the NDV VLPs were non-fusogenic, and therefore incapable of inducing syncytia formation.

Here we describe NiV VLPs composed of the two surface glycoproteins G, and F, and the matrix protein M. The G and F proteins were included because they mediate attachment and entry into the host cell [50], [51], [52], both are major targets of neutralizing antibodies, and both are major players in vaccine induced protection [52], [53], [54]. NiV G and F together are also the most effective as immunogens; this was elucidated in a canary pox virus vector-based experimental protective efficacy study [26]. The M protein was included in our formulation because it is required for particle formation and release [20], [25], [45]. Under optimized conditions, we were able to make substantial, quantifiable amounts of NiV VLPs composed of these three NiV proteins. This has allowed us to characterize their properties in detail to show that they possessed many virus-like/vaccine desirable properties *in vitro*. It has also allowed us to test for immunogenicity *in vivo* in Balb/c mice; note that although NiV does not cause disease in these animals, NiV proteins injected in them are known to induce robust neutralizing antibody response [29], [33], [34]. Importantly, NiV-specific mouse monoclonal antibodies are protective in the hamster model of NiV disease [55].

In this study, careful assessment of immunogenicity has shown for the first time, that these NiV VLPs are able to induce neutralizing antibody response. We have also provided a detailed methodology to optimize production of the VLPs for research purposes. Beyond this, we have provided the first CryoEM study of NiV VLPs and thus provide a careful assessment of their morphology. We further demonstrate that NiV VLPs can trigger “fusion from without” upon addition to cells. To our knowledge this is a first for an enveloped VLP. Finally, we have shown that NiV VLPs activate innate immune signaling in “infected” cells and provide a transcriptional profile of this response. Based on all these attributes, NiV M, F and G-protein-containing VLPs show promise as vaccine and will be the basis for undertaking future protective efficacy studies in animal models of NiV disease.

## Materials and Methods

### Protein expression vectors, cells and viruses

NiV expression plasmids pCAGGS- G, F, and M are all under the control of chicken beta actin promoter [56], and they were constructed in the laboratory of one of the co-authors of this study (CB) as described previously [20]. Human embryonic kidney 293 cells (ATCC, CRL-1573) and 293T cells (ATCC, CRL-11268) were grown in Dulbecco's minimum essential medium supplemented with10% fetal bovine serum (FBS) and penicillin and streptomycin, and maintained in the same medium containing 2% FBS. The minigenome that was used for optimizing VLP formation has been described previously [57]. All the initial minigenome-based optimization steps were done in BHK-T7 cells (a gift from Dr. N. Ito). The same conditions were applicable to produce VLPs in 293T cells and they were used throughout to generate the VLPs used for the work described in this study.

### Transfection

293T cells were grown in Dulbecco's complete medium to achieve semi-confluent (80–90% density) cell monolayers. The cells were transiently transfected with the plasmids constructs using the lipid reagent Lipofectamine 2000 according to the general guidelines provided by the manufacturers' instructions (Invitrogen Inc). At 48 hrs post-transfection, the VLP-containing cell supernatants (SUP) were harvested for concentration and purification of the VLPs. Because of the fusogenic property of our VLPs, there was widespread syncytia formation at this time point although the cells were still adherent.

### VLP harvest and purification

VLPs released in the transfected-cell SUP were harvested and clarified by centrifugation at 3,500 rpm for 30 minutes at 4°C and concentrated by sucrose density gradient centrifugation based on previous descriptions [44], [45], [58]. Briefly, the clarified SUPs were concentrated by ultracentrifugation through 20% sucrose cushion in TN buffer (0.1 M NaCl; 0.05 M Tris-HCL, pH 7.4) at 200,000× g for 8 hours at 4°C. The resulting VLP pellet in ∼0.5 ml volume was purified on a discontinuous sucrose gradient formed by layering 80%, 65%, 50% and 10% sucrose in TN buffer. After centrifugation at 186,000× g for 8 hours, the top ∼1.5 ml of the gradient (which included the VLP-containing band at the interface between the 10% and 50% sucrose layers) was resuspended in 20% sucrose buffer and centrifuged once more at 160,000× g for one hour. The resulting pellet was resuspended in 20% sucrose solution in endotoxin-free TN bufffer and stored at 4°C for subsequent analysis. Supernatant of 293T cells transfected with empty pCAGGS plasmid and processed similarly (referred to as “mock” particles) served as negative control when needed.

### VLP infectivity assay

Since the ratio of the protein expression plasmids used at transfection and the time of harvest may have a bearing on the level of VLP formation, a minigenome-based *VLP infectivity assay*, similar to those described previously [59], [60] *was* used to determine the relative concentrations of the constituent plasmids, and to determine the kinetics of VLP formation for optimal production. This assay provides only a comparative assessment of VLP formation since it only accounts for VLPs that are able to incorporate and passage minigenomes. However, based on the assumption that the ratio of empty and minigenome-containing VLPs will be equivalent in each reaction, the method provides an indirect means to determine the optimal set of conditions for VLP production as determined by VLP-incorporated minigenome-encoded CAT enzyme activity. Briefly, the steps involved in the VLP infectivity assay were 1) transfection of NiV minigenome construct and co-transfection with full complement of the NiV protein expression plasmids, N, P, L, M, F and G, using Lipofectamine 2000. 2) following replication (48 hours post-transfection), passage of equal volume of VLP-containing transfected cell SUP on to fresh cells previously transfected with N, P and L plasmids and 3), determination of CAT activity in the VLP infected cells 48 hours later. Replication of the VLP-incorporated incoming mingenomes based on reporter gene activity indicates the level of particle formation and release, VLP infectivity, and successful minigenome packaging.

### CAT assays

FAST CAT Assay kit (Molecular Probes) was used according the manufacturer's instructions and allowed accurate quantification of CAT enzyme levels over a wide linear range.

### Electron Microscopy (EM)

VLPs were purified as described. The particles were adsorbed on Formvar carbon coated copper grid by floating it on a drop of VLP suspension for 15 minutes, the grids were blotted, and then negatively stained with 2% aqueous uranyl acetate for viewing by transmission electron microscopy.

### Cryoelectron (CryoEM) microscopy

The VLPs were vitrified as reported previously [61] on holey carbon film grids (C-flat™, Protochips, Raleigh, North Carolina). VLPs were imaged at 40,000x indicated magnification using a 4k×4k slow-scan CCD camera (UltraScan 895, GATAN, Inc., Pleasanton, CA) using a low-dose imaging procedure.

### Immunogold labeling

Unfixed VLPs were used for immunogold labeling to limit antibody reactivity to the cell surface proteins. The particles were adsorbed on formvar coated nickel grids, stained with NiV specific primary antibody (hyper immune mouse ascites fluid, HMAF, obtained from Dr. P. Rollin, CDC) diluted in buffer (1% BSA in 0.05 M tris buffer) rinsed in wash buffer (0.1% BSA in 0.05 M tris buffer), stained with colloidal gold labeled goat anti-mouse secondary antibody (Jackson ImmunoResearch Laboratories), washed, and then negatively stained with 2% uranyl acetate for viewing by EM.

### VLP Protein concentration

The total protein concentration of the purified VLP preparations was measured by the BCA (Bicinchoninic Acid) method (Thermo Scientific Laboratories).

### Western blotting

VLP composition was determined by western blot analysis. Briefly, purified VLPs resuspended in endotoxin free PBS were lysed by resuspending them in equal amount of 2x SDS protein-loading buffer and loaded into a 12% SDS-polyacrylamide gel with a 4% stacking gel. 293T cell lysates processed similarly were run in parallel as negative cell control. Following electrophoresis to resolve the protein bands, and transfer to membrane, the blot was incubated with NiV-specific HMAF primary antibody at a dilution of 1∶1000 dilution, overnight at 4°C, and HRP-conjugated anti-mouse secondary antibody (from GE Healthcare) at a 1: 20,000 dilution for one hour at room temperature. The proteins were revealed using western blot detection reagents according to instructions provided by the manufacturer (GE Healthcare).

### Protocol to immunize Balb/c mice

These studies were undertaken with the approval of the Institutional Biosafety Committee (Protocol# #01/08-2010-1) and the Institutional Animal Care and Use (IACUC) Committee (Protocol # 0904028). Five to six week old female Balb/c mice (Harlan Laboratories) were housed in microisolater cage for 4 days in the Animal Resource Center at the University of Texas Medical Branch before beginning the immunization protocol. Mice in groups of five were immunized by subcutaneous inoculation of four different concentrations of VLPs (1.75, 3.5, 7 or 14 µg/mouse, referred to subsequently as treatment groups A through D respectively) prepared just prior to use in sterile endotoxin free PBS. No adjuvant was used. A group of five mice inoculated with sterile endotoxin free PBS served as negative control group. Mice in the four treatment groups (A through D) were boosted (6 µg/mouse) on days 15 and 29; the negative control group received PBS. Blood was collected from the submandibular vein of the animals on days −1, 14, 21, 28 and 35; they were euthanized on day 35.

### VLP-induced immune response

#### Plaque Reduction neutralization test (PRNT)

Two-fold dilutions of test sera were made in 50 µl cell culture medium. Under biohazard level 4 conditions, each of the diluted sera were mixed with 50 µl of NiV diluted to generate ∼30 plaque forming units and incubated for 30 min at 37°C. The pre-incubated virus-antibody mix was added to Vero cell monolayers grown in 96 well plates and incubated for 30 min at 37°C when the inoculum was removed and replaced 150 µl of cell media. After incubation at 37°C for 24 h, the cells fixed in 100% ice-cold methanol and staining by indirect immunofluorescence assay as follows: The wells in the plate were blocked with BSA/PBS and stained with rabbit sera raised against the G protein of HeV, and goat anti-rabbit Alexa Fluor 488 conjugate (Invitrogen) diluted 1∶1000 in blocking buffer. Viral plaques were visualized and counted, and neutralizing antibody titers were reported based on reduction in plaque count by 50% relative to the untreated control (PRNT_50_).

#### Antibody levels measured by Immunofluorescence assay (IFA)

For IFA, NiV-specific total antibody levels were measured by using NiV G, F and M expressing 293T cells as target antigen. Thirty six hours post-transfection, the cells were harvested, fixed in paraformaldehyde, cytospun (Cytocentrifuge, Thermoscientific) on glass slides to obtain monolayered preparations and then stored at 4°C, and used as antigen within three weeks of preparation. On the day of use, the slides were washed in PBS, permeabilized with Triton-X-100 and blocked with BSA/PBS. After incubation with two fold dilutions of the test sera, the cell monolayers were washed and stained with Alexa fluor 488-conjugated goat anti-mouse antibody according the manufacturer's (Molecular Probes) instructions. Negative and positive controls were run in parallel with each batch.

### Gene expression profile by Real-time PCR

VLP-mediated transcriptional activation was tested for eighty four genes involved in Toll-like receptor (TLR)-mediated signal transduction using RT^2^ Profiler PCR array (SABiosciences). The 96 well array format included mediators of TLR signaling including adaptors and proteins that interact with the TLRs, and members of NFKB, JNKp38, NF/IL6 and IRF signaling pathways downstream of TLR signaling. Briefly, 293 cells grown overnight in 60 mm dishes were exposed to 10 µg of purified VLPs suspended in 1 ml of OPTI-MEM (Invitrogen). “Mock particles” (see Methods, VLP harvest and purification) resuspended similarly and exposed to 293 cells served as negative control. The inoculums were adsorbed on the cell monolayers for 3 hours at 37°C when additional 1.5 ml of OPTI-MEM was added and the dishes further incubated. Twenty fours post VLP exposure, total cell RNA was extracted according to the manufacturer's (SABiosciences) instructions. The integrity of the RNA was verified by agarose gel electrophoresis and the same concentration of total cell RNA from the VLP-stimulated and “mock” stimulated cells were used for gene expression profiling by Real-time PCR using Eppendorf Mastercycler unit. The array plate included positive and negative controls for quality assurance, and three sets of housekeeping genes for normalization for data analysis. The fold-change in gene expression in the VLP stimulated 293 cells relative to the “mock” stimulated 293 cells was calculated by the ΔΔCt method according to the manufacturer's instructions.

## Results

### Optimization of conditions for the production of NiV VLPs

In preliminary studies it was found that co-expression of NiV G, F and M proteins in 293T cells resulted in the formation of VLPs that bud out into the transfected cell SUP and that they can be harvested, concentrated and purified as described under Methods. However, the VLP yield was low. To improve the efficiency of VLP formation we proceeded to optimize the ratio of the three expression plasmids used at transfection. We speculated that this would be important based on the fact that a), during replication, paramyxoviruses form a transcription gradient where the 3′ proximal genes are transcribed more abundantly than the successive downstream genes [52] and b), the stoichiometry of interaction of the viral proteins has proved to be critical in plasmid-driven minigenome and full-length rescue systems [62].

The importance of protein ratios for VLP formation was alluded to in a previous NDV study where the expression plasmids were co-transfected at “pre-determined concentrations” to produce VLP-incorporated protein ratios analogous to those in virus infected cells [49]. In a study by Patch el al [48], equivalent amounts of NiV N, M, F and G were initially used to produce the VLPs. In that study, VLPs were subsequently also made by adjusting NiV expression plasmid concentrations by experimental variations similarly to that in the NDV study [49]. The efficiency of particle formation and budding in both these, and many other paramyxovirus VLP formation systems was based on M protein release [43], [44], [45], [46], [48].

**Figure 1:**
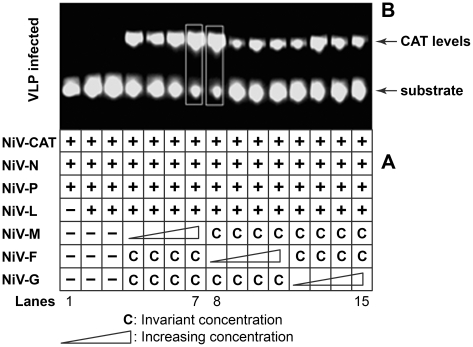
The amount of M, F and G plasmids used at transfection has a bearing on the level of particle formation based on minigenome-encoded reporter gene levels in VLP-infected cells. Cells were transfected with increasing concentrations of M, or F or G expression plasmids (indicated by a triangle) while keeping the concentration of the other two plasmids fixed. They were co-transfected with the previously optimized minigenome and N, P and L constructs [16], [57]. Forty eight hours post transfection, the cell SUPs were clarified by centrifugation, and same volume of SUP from each sample was used to infect new cell monolayers (VLP infected) which were transfected 24 hours previously with the core plasmids N, P and L to support replication of the VLP-incorporated minigenome RNA; the VLP-infected cells were harvested 48 hours later for reporter gene analysis. **Panel A** shows the plasmids transfected in each reaction. **Panel B**: shows minigenome-encoded CAT activity in VLP infected cell monolayers. Lane 1 is a negative control. Absence of CAT activity in duplicate lanes 2 and 3 indicates that VLP formation, and consequently VLP-incorporated minigenome transfer and expression, does not occur in the absence of M, F and G proteins. The results in lanes 4 through 15 shows that CAT levels varied in VLP infected samples depending on the concentration of M, F and G constructs used at transfection. Thus, the amount of M, F and G plasmids used at transfection had a bearing on the level of particle formation, and the consequent CAT reporter gene transfer and expression. CAT activity in the VLP infected reactions appeared optimal in the boxed lanes 7 and 8. Further analysis of CAT levels in the linear range (data not shown) demonstrated that optimal VLP formation was achieved with the ratios of M, F and G expression plasmids of 3∶1∶1 (lane 7).

We have chosen a minigenome-based functional assay, the VLP infectivity assay (described under Materials and Methods), to determine optimal expression plasmid ratios for efficient VLP formation based on reporter gene readouts. Briefly, 293T cells were transfected with plasmids as shown in Figure 1. For titrating NiV G, F and M plasmids, increasing concentrations of either G, or F or M expression plasmids were, in turn, co-transfected with fixed concentrations of the other two plasmids. The minigenome and N, P and L plasmids were transfected using a predetermined ratio [57]. The VLP-containing cell SUP was harvested 48 hours post-transfection, clarified by centrifugation, and equal volume from each was passaged onto fresh cell monolayers (VLP-infected cells) previously transfected with the core proteins required to support the incoming packaged minigenomes. The *VLP infected cells* were harvested 48 hours later and tested for optimal particle production based on incoming minigenome-encoded CAT activity. This time point was chosen because maximal VLP formation was also found to be time dependent and optimal at 48 hours post-passage (data not shown). The reproducibility of the results was verified in an independent repeat experiment. Results presented in Figure 1 show that within the given range, and based on the levels of minigenome-encoded CAT activity, varying the concentrations of G, F and M plasmids had a bearing on VLP formation. CAT activity in the VLP infected cells appeared optimal in the boxed lanes 7 and 8 but further analysis to ensure reporter activity in the linear range (data not shown) indicated that the largest amount of minigenome-containing NiV VLPs were produced when the cells were transfected with the NiV M, F and G plasmid ratios of 3∶1∶1 as in lane 7. This ratio was used for making all our VLP preparations.

### Morphologic similarity between NiV VLPs and the authentic virus

**Figure 2:**
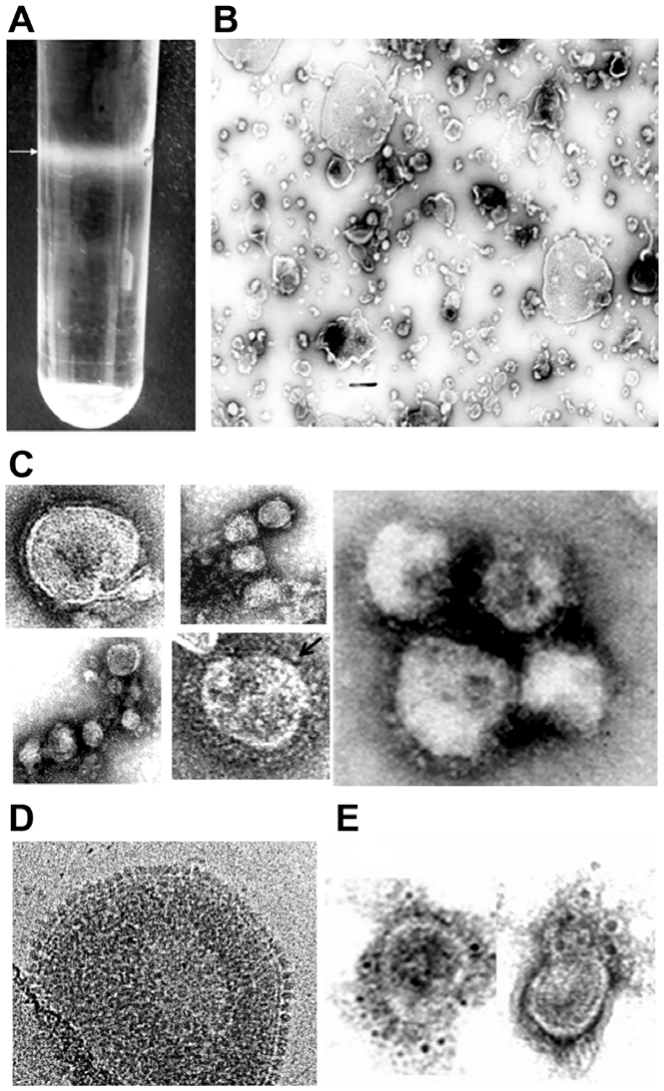
Co-expression of NiV proteins G, F and M results in the formation substantial quantities of VLPs morphologically resembling NiV virions. VLPs released in the transfected cell-supernatant were harvested and purified as described under Methods, and viewed by EM and cryoEM to evaluate their morphology. Under optimized conditions, substantial amounts of VLPs were produced, (**A**) shows VLP-containing band in the sucrose gradient. Negatively stained sample in (**B**) show numerous well preserved VLPs. Selected VLPs which were magnified (**C**) to show clearly the spikes of the glycoproteins present on the VLP surface; an occasional particle had what appeared to be a double fringe (shown with an arrow), a feature normally thought to be associated with *Hendra virus* particles [9]. (**D**) Shows cryoelectron micrograph of one of our VLPs. The glycoprotein spikes and their spatial arrangement are seen here even more clearly. (**E**) Shows functional assembly and immunoreactivity of NiV glycoproteins at the VLP surface. Unfixed particles were stained by immunogold labeling technique using NiV-specific polyclonal antibody and gold labeled secondary antibody. Unfixed particles were used so that only the surface proteins would be available for immunoreactivity. The Figure shows two VLPs with gold-decorated proteins on the VLP surface.

The optimized conditions were applied to transfect G, F and M expression plasmids in 293T cells grown in 10 cm dishes. The VLP-containing culture SUPs were harvested 48 hours later, and concentrated and purified as described. Briefly, the clarified SUPs were concentrated by ultracentrifugation through 20% sucrose cushion, and then purified on a discontinuous sucrose gradient. The VLP pellet was resuspended in TN buffer and viewed by EM after negative staining. The result presented in Figure 2A shows a VLP-containing band in the sucrose gradient. Viewing of the negatively stained purified particles by transmission electron microscopy (Figure 2B) showed numerous virus-like particles. The size variation of these VLPs was consistent with the parental virus: NiV is a pleomorphic virus ranging in size from 40–1900 nm [63], [64]; the sizes of the VLPs ranged from ∼40–500 nm. The particles also resembled authentic NiV morphologically, and this is seen more clearly in the magnified images presented in Figure 2C; here, the fringe of the glycoproteins is clearly visible on the VLP surface. An occasional VLP had what appeared to be a double fringe (shown with an arrow), a feature more frequently associated with *Hendra* rather than NiV virus particles [64]. The image in Figure 2D is a cryoelectron micrograph of one of our VLPs; the overall surface appearance is virus-like, which is described as dense, ordered and repetitive [31], and it shows the surface glycoproteins and their spatial arrangement even more definitively.

### Identification of NiV-specific proteins in the VLPs

**Figure 3:**
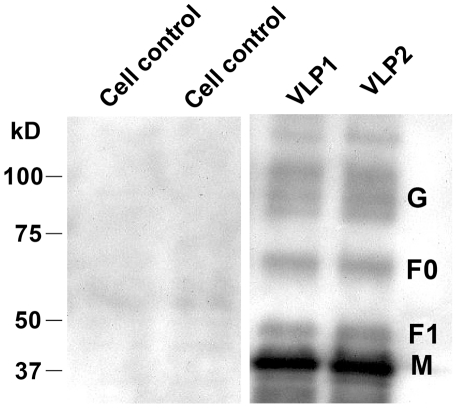
VLP-incorporated NiV proteins. The Figure shows western blot analysis of NiV VLPs to verify their composition. The VLPs were processed and analyzed by SDS-PAGE as described using manufacturer's instructions. VLP protein bands corresponding in size to NiV proteins G, F_0_, F_1_ and M were clearly visible.

To verify whether the NiV proteins were incorporated into the VLPs as designed, purified particles were analyzed by western blotting using NiV-specific mouse antibody, and HRP-conjugated anti-mouse secondary antibody as described under Methods. The right hand panel in the Figure 3 shows VLP-incorporated proteins in two different preparations of NiV VLPs. The protein bands are consistent in size to NiV proteins G, F_0_, F_1_ and M proteins [17], [48]. The relative amounts of the VLP-incorporated G and M proteins appeared to be similar to that reported in NiV virions also [17]. This was in spite of the fact that the viral proteins in that study were revealed using rabbit sera raised against bacterially expressed *Hendra* virus proteins. However, the ratio of the VLP-incorporated F_1_ to F_0_ was different from that in the virions. This difference is more likely to be a reflection of timing and protein turnover rather than the reagents used to reveal them since in a previous study, pulse chase experiments have shown that similarly to the VLP-incorporated F_1_ to F_0_, intracellular cleavage of the precursor NiV fusion protein by cathepsin L results in near equal mix of mature fusogenic, and the precursor forms [65]. Absence of the NiV-specific bands in two different 293T cell lysate preparations processed similarly (and shown in the left hand panel in Figure 3) confirms specificity of the VLP-incorporated proteins.

### Immunoreactivity of VLP surface glycoproteins

The immunoreactivity of the VLP surface glycoproteins was verified by staining purified unfixed VLPs by the immunogold labeling technique using NiV-specific mouse antiserum and 6 nm colloidal gold particle-conjugated goat anti-mouse secondary antibody (Jackson ImmunoResearch Laboratories Inc). The particles were viewed by EM after negative staining. The use of unfixed particles assured that only the surface-exposed antigens would be reactive. Numerous VLPs with the gold particles decorating their surface were seen; Figure 2E shows two such VLPs.

### Inhibition of VLP-induced syncytia formation by NiV-specific antibodies

**Figure 4:**
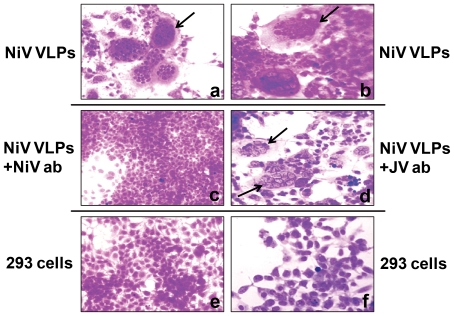
NiV VLP-induced syncytia in 293 cells is blocked by prior treatment with NiV-specific antibody. NiV VLPs were pre-incubated for one hour at 37°C with either NiV specific antibody or Junin virus-(JV) specific antibody, or with OPTI-MEM I medium only (untreated VLPs) before inoculating onto 293 cell monolayers grown overnight in 60 mm dishes. The plates were incubated overnight at 37°C and stained with crystal violet. The results show VLP-mediated formation of syncytia (a and b) that were blocked (c) when the VLPs were pretreated with NiV-specific antiserum but not blocked (d) when the VLPs were pre-treated with Junin virus-specific antibody. Images e and f show uninfected 293 cells. Arrow points to syncytia.

Syncytium formation is a classical feature of NiV and other paramyxovirus-induced cytopathology that can be blocked by virus-specific neutralizing antibody. A similar observation was made when 293 cells were “infected” with the NiV VLPs. Briefly, the VLPs were pre-incubated with NiV-specific antibody, Junin virus (JV)-specific antibody, and with OPTI-MEM I (Invitrogen Inc) medium only for one hour at 37°C before inoculating onto near confluent 293 cell monolayers grown overnight in 60 mm dishes. The inoculum was removed after incubation for 3 hours at 37°C, replaced with OPTI-MEM I, and the plates were further incubated overnight at 37°C overnight. The monolayers were then viewed for the formation of syncytia after staining with crystal violet. The results in Figure 4 show that 293 cells exposed to NiV VLPs induced syncytium formation and that this process was neutralized by NIV-specific antibodies; prior incubation with the unrelated JV antibodies failed to block this process.

### NiV VLPs as immunogens in Balb/c mice

**Figure 5:**
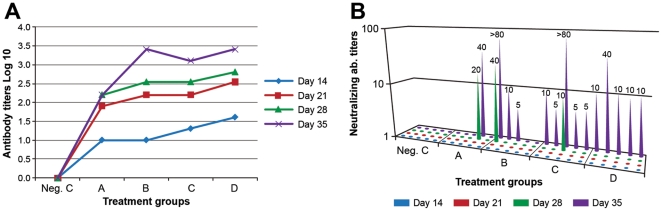
NiV VLP-induced immune response in Balb/c mice. NiV-specific antibody levels of serum samples from mice immunized subcutaneously three times were measured by IFA and by Bio-Plex microsphere methods. Neutralizing antibody response was evaluated by PRNT_50_. The experiments were done in duplicate. **A**: For evaluation by IFA, sera from each treatment group were pooled for analysis. The results show serocoversion for each of the four treatment groups. In general, the titers increased progressively with time and with the VLP dose although by day 35, similar titers were seen with the three higher VLP doses. **B**: Shows neutralizing antibody titers (PRNT_50_) in sera from each mouse collected on the stated days. Neutralizing antibodies were seen starting on day 28 after primary inoculation. The response was again clearly dose dependent; all mice in the two highest treatment groups (C and D) showed neutralizing response by day 35. Such response was seen in 3 of 5 and 1 of 5 mice in the two lower (B and A respectively) treatment groups.

Mice in groups of five were inoculated subcutaneously with four different concentrations of purified VLPs and boosted as described under Methods. The negative control group of five mice were inoculated with sterile endotoxin free PBS for each inoculation. The mice were bled from the submandibular vein on the day before primary inoculation, and then on days 14, 21, 28 and 35. For initial evaluation, sera from each treatment group were pooled, and the IFA method used to determine levels of NiV-specific antibodies. The results in Figure 5A show that titers (reciprocal of the highest serum dilution showing reactivity) increased with time post primary inoculation, i.e., the highest titers (1∶2560) were seen on day 35. Titers also increased with VLP dosage although by day 35, the three higher treatment groups seemed to produce similar titers. As expected, the mice in the negative control group remained nonresponsive.

*All* sera were tested individually by plaque reduction neutralization method by doubling dilution of each sample (1∶5 to 1∶80) as described under Methods. The results (Figure 5B) showed distinct association between VLP dosage and the ability to mount a neutralizing antibody response. Mice inoculated with the two highest VLP doses (treatment groups C and D) were each able to induce neutralizing antibodies by day 35. When samples from mice receiving the two lower concentrations of VLPs (3.5 ug/dose and 1.75 ug/dose, corresponding to treatment groups B and A respectively) were similarly tested, 3 of 5 and 1 of 5 mice respectively induced neutralizing antibody response; the titers ranged from 1∶5 to >1∶80. As expected, the control mice did not induce neutralizing response.

### NiV VLP-induced activation of genes involved in signaling innate immune response

**Figure 6:**
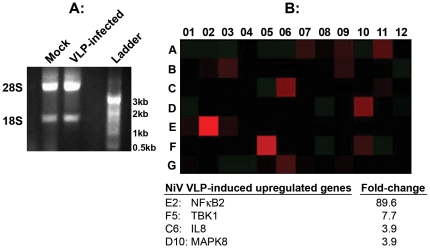
VLP-induced modulation in transcription profile of genes involved in signaling of innate immune response in 293 cells by PCR Array. 293 cells were grown overnight in 60 mm dishes and were infected with 10 µg of purified VLPs suspended in OPTI-MEM (Invitrogen). Mock infected cells served as negative control. The inoculum was adsorbed on the cell monolayers for 3 hours at 37°C when it was supplemented with fresh OPTI-MEM and further incubated overnight when total cell RNA was extracted according to the manufacturer's (SA Biosciences) instructions. **A**: shows the integrity of the RNA used for this analysis. Note the ∼2∶1 ratio of 28S:18S which is a good indication of the integrity of the RNA. Equal concentration of the RNA from the mock and VLP-exposed cells was used for expression profiling by RT^2^ PCR Profiler PCR Array according the manufacturer's (SABiosciences) instructions. **B**: Shows the heat map, it is a visual illustration of the relative expression levels in the VLP-stimulated vs. the “mock” stimulated control cells of the all the genes in the array: The four genes differentially expressed by a factor of ∼4 fold or greater (shown as red squares) are listed below the heat map.

A PCR array format (SABiosciences) was used to investigate modulation in transcription profile of 84 genes involved in innate immune responses to include TLR signaling family and members of the downstream signaling pathways, NFKB, NF/IL6, IRF and JNKp38. These genes represent key sensors of non-self that signal, and ultimately shape the nature of innate immune response that modulates the type and duration of adaptive immune responses [66], [67]. The differential expression of genes in VLP-exposed 293 cells relative to the “mock” infected 293 cells was measured by real-time PCR. Same concentration of total cell RNA from the VLP-stimulated and the “mock” stimulated control cells were used for first strand synthesis and Sybr green PCR amplification of the relevant genes as described under Methods. The integrity of RNA in each sample was confirmed by gel electrophoresis (Figure 6A). Data representing the differential transcription profile of VLP exposed vs. “mock” stimulated cells is shown as a heat map (Figure 6B). A 4-fold cutoff threshold was used to determine modulation in gene expression. We noted significant VLP-stimulated up-regulation (89 fold and 7 fold) in the expression of NFKB2 and TBK1 genes respectively. Close to four fold (3.9 fold) up-regulation was noted also in IL-8 and MAPK8 genes. NFKB2 and IL-8 are target genes in the downstream NFKB pathway, and TBK1 which are in the IRF and JNK/p38 pathways respectively.

## Discussion

Using a minigenome-based functional assay, we have established conditions (described under Results and shown in Figure 1) that have allowed us to produce substantial quantities of NiV VLPs to be able to undertake the studies described in this manuscript. We have shown that these particles are functionally assembled, biologically active and are able to induce innate immune responses, and a neutralizing antibody response. Native VLPs have been used to study various aspects of the virus lifecycle, as carriers to deliver heterologous proteins for vaccination, and to deliver small molecules for gene therapy purposes. Particularly importantly, they have been used highly effectively as vaccines in their native form [31], [32], [39], [40], [41].

No vaccine for NiV disease has been developed so far that would be both safe and protective for humans. The two vaccination strategies that have already been explored are the canary pox-based vector approach [26] and soluble subunit approach [28], [29]. NiV vaccine by the former method is undergoing development as a veterinary vaccine [26]. The same approach is being evaluated for human use vaccines, mainly for the prevention of HIV and AIDS [27]. The subunit approach has limitations as already mentioned above [28], [29], [30], [31], [32]. One particular challenge revealed by studies that tested a soluble NiV G protein-based subunit vaccine formulated with adjuvant is the potential difficulty of eradicating infection in the central nervous system. In that study [28], live virus was present in the brain of one cat, and viral RNA was present throughout the 21 day post-challenge period in the brains of the remaining challenged animals. A recently reported vaccination strategy [34] requires simultaneous inoculation of two VSVΔG vectors, one expressing NiV G, and the other expressing NiV F proteins. It was of interest to note that supernatants of cells co-infected with these two defective viruses were infectious and could be passaged indefinitely in the absence of VSV G trans-complementation. This vaccination approach seems promising since self-propagated stock of these two viruses induced robust neutralizing antibody response in mice. However, potential pathogenicity of VSV-based vaccine vectors remains a concern [34], [35]. The potential of a recombination event resulting in a single VSV vector virus expressing both these NiV proteins is unlikely, but it may still be problematic for a human use vaccine.

Native VLPs like the ones we have produced allow the viral proteins to be presented to the immune system in the same conformation as in the virion for effective B and T cell response [31]. VLPs are particularly effective in producing a protective antibody response because of their virus-like size range, their particulate nature, and their virus-like dense, repetitive and ordered surface structure [31], [36]. The spacing of the antigenic epitopes on the VLP is also optimal for B cell activation [31]: EM analysis showed that our particles resembled the real virus in terms of size and surface structure [63], [64]. The image in Figure 2D is the first elucidation of VLP structure of any paramyxovirus imaged by CryoEM, and it provides a careful assessment of their morphology; it alludes to a surface similar to that revealed for measles virus by the same imaging technique (Dr. Elizabeth Wright, Emory University). The proteins on the VLP surface are clearly visible here; the average distance between the spikes was 9.13 nm and standard deviation was 1.72 nm. This is of interest given that epitopes spaced between 5 and 10 nm are known to be sufficient to drive optimal B cell activation [36].

NiV M, F, G and N protein-containing VLPs consistent in size and morphology to the parental virus have also been reported in a previous study which evaluated protein-protein interaction that facilitate VLP formation [48]. However, in that study, most of the particle-incorporated NiV F protein was predominantly in the uncleaved precursor form. This finding is clearly distinct from ours since our VLPs contained substantial amounts of cleaved F protein, and this may have been related to ratios of the interacting proteins expressed in 293T transfected cells. In a recent study of NDV VLPs [49], the particle-incorporated proteins were reported to have virus-like protein ratios, but the F protein remained in its precursor form because the cleavage site required to produce the fusion competent form was mutated by design. What effect a VLP-incorporated non-fusogenic F protein may have, relative to the fusogenic form, on the level and quality of VLP-induced immune response is not clear at present since difference in immunogenicity between fusion-competent and fusion-defective VLPs has not been experimentally evaluated so far. However, a recent report suggests that viral fusogenic membrane glycoproteins may enhance vaccine potency [68].

Immunogold labeling of our unfixed NiV VLPs confirmed that the surface proteins in our VLPs were functionally assembled and they were biologically active (Figure 2E). We could deduce the presence of biologically active G and F proteins on the VLP surface by the fact that they were able to induce the formation of syncytia in 293 cells (Figure 4); this is a process that requires the interaction of both the surface glycoproteins, the attachment protein G, and the fusion competent F protein, when they come in contact with the cognate receptor-bearing cells. Formation of syncytia or multinucleated cells in replication competent enveloped viruses, especially paramyxoviruses, is induced by a process that is described as “fusion from within”, and it can be blocked or neutralized by prior treatment of the virus with specific antisera. In contrast, “fusion from without” is induced by non-replicating viruses at high multiplicities of infection, and it too can be blocked by pretreatment with virus-specific antibodies ([69], and references therein; [70]). Our non-replicating particles likewise induced syncytia formation in 293 cells that could be neutralized with NiV-specific antibodies (Figure 4). To our knowledge, this is the first study describing fusion from without induced by VLPs of any paramyxovirus, or any other enveloped viruses, although it has been described for the VLPs of the non-enveloped rotavirus [71]. The mechanism(s) of fusion from without is not clear but two models have been proposed [72], [73]; one proposes that particles connecting adjacent cells effectively promote fusion between them, and the other is that when particles decorated with the surface glycoproteins fuse with the target cell membrane, the glycoprotein complexes diffuse freely in the lipid bilayer, and mimic fusion from within. The type of VLP-induced syncytia formation and eventual cell death is also not known. We are in the process of investigating it.

Neutralizing antibody response is the critical correlate of protection mediated by prophylactic vaccines [53], [54] and native VLPs promise to be highly effective prophylactic vaccines for paramyxoviruses like NiV, and others like NDV and measles where neutralizing immune response is known to play a pivotal role in protection against disease [53], [54], [55], [74]. Our VLPs were highly effective immunogens, and all, especially in the three higher treatment groups produced high levels of response by day 35 (Figures 5A). Importantly, NiV VLPs were able to induce neutralizing antibodies. This response was clearly dose-dependent (Figure 5B). All ten mice receiving a primary inoculation of 7 or 14 µg VLPs (subgroup C and D) were able to produce such response; but even of those animals that received a first dose of only 3.5 or 1.75 ug/mouse (treatment group B and A respectively), 3 of 5 and 1 of 5 produced neutralizing antibodies. Neutralization antibody response was first seen on day 28, and increasing titers were seen in some animals within a week of it; we believe that this response, induced by our non-replicating and potentially safe particles, *formulated without adjuvant*, compares favorably with the levels of such response induced at an equivalent time point by some replication competent pseudotype viruses [34].

Immunogenicity to native VLPs has been reported previously for one other paramyxovirus namely NDV [49]. In that report, immune response to NDV VLPs was evaluated by primary inoculation of mice intraperitoneally with VLP concentrations ranging between 10 and 40 µg, and a booster dose of 10 µg, without adjuvant. NDV-specific titers by ELISA were high in each mouse in each treatment group. Neutralizing antibody response to 20 and 40 µg of these particles was also detected.

The nature of innate immune response dictates the type and duration of adaptive immune response [67], [75]. The mechanism by which NiV VLPs are recognized by host cells and trigger the induction of innate immune response, and how this translates into effective adaptive immunity is not known. Here we have taken the first step (Figure 6) towards understanding this process. With the experimental conditions as described, we observed VLP-induced activation of some of the genes that are known to be involved in the induction of an effective innate immune response [75]. Results presented in the heat map in Figure 6 show that relative to the “mock” treated cells, NFKB2 gene (in the NFKB pathway) was up-regulated 89 fold as a result of VLP exposure, and TBK1 (in the IRF pathway) was 7 fold higher. In the light of these findings, we are testing PCR array expression profiles of the same set of 84 genes in 293 and other cells at earlier and later time points to identify their upstream effectors, and NiV VLP-responsive signaling networks. In this respect, the murine system, with the many available immunological reagents and knockout strains may provide the best system to identify these host sensors. Currently there is minimal information on live NiV infection-responsive cell-signaling changes [76] and there is none on array-based transcriptional alterations for comparative analysis. Likewise, it has not been possible to compare the NiV VLP-induced transcription modulation with those induced by other paramyxovirus VLPs since to our knowledge, such studies have not been undertaken so far. Lastly, a growing number of reports point to viral surface glycolproteins as relevant in host cell signaling and triggering of innate immune response. We believe that particles like NiV VLPs, with many virus-like properties (including their surface glycoproteins organized to resemble the parental virus, Figure 2D) would induce an effective innate immune response for the promotion of the desired adaptive immunity [67], [76].

Finally, as described above, our VLPs were highly effective as immunogens, able to induce neutralizing antibody response in all animals with primary inoculation of as little as 7 µg VLP protein each. Fusogenic property of our VLPs may be critically relevant in this regard in the light of recent findings, and would need to be experimentally verified by comparing the potency of fusion-competent and fusion-defective VLPs as vaccine [68].

In conclusion, we have been successful in producing substantial quantities of NiV VLPs needed to characterize NiV VLPs, we have demonstrated their many virus-like properties, and their effectiveness as immunogens in Balb/c mice. These findings are the basis on which we will be undertaking future challenge studies in the hamster model of NiV disease [77].
